# Does carbohydrate supplementation enhance tennis match play performance?

**DOI:** 10.1186/1550-2783-10-46

**Published:** 2013-10-22

**Authors:** Rodrigo Vitasovic Gomes, Caroline Dario Capitani, Carlos Ugrinowitsch, Michael Christopher Zourdos, Jaime Fernandez-Fernandez, Alberto Mendez-Villanueva, Marcelo Saldanha Aoki

**Affiliations:** 1 School of Physical Education and Sport, University of São Paulo, Av Prof Mello Moraes 65, São Paulo, SP 05508-030, Brazil; 2 School of Applied Sciences, University of Campinas, Av Pedro Zacaria, 1300, Limeira, SP 13484-350, Brazil; 3 Department of Exercise Science and Health Promotion, Florida Atlantic University, 777 Glades Road, FH 11 Boca Raton, FL 33431, USA; 4 Sports Research Centre, Miguel Hernandez University, Avda. Universidad s/n, Elche, Alicante 03202, Spain; 5 ASPIRE Academy for Sports Excellence, Al Waab, Doha 22287, Qatar; 6 School of Arts, Sciences and Humanities, University of São Paulo, Av Arlindo Bettio, 1000, São Paulo 03828-000, Brazil

**Keywords:** Match analysis, Glycemia, Performance

## Abstract

**Background:**

Carbohydrate (CHO) ingestion may be an interesting approach to avoid significant decrement to the tennis match performance. The aim of the present investigation was to assess the effects of CHO supplementation on tennis match play performance.

**Methods:**

Twelve young tennis players (18.0 ± 1.0 years; 176 ± 3.4 cm; 68.0 ± 2.3 kg; body fat: 13.7 ± 2.4%) with national rankings among the top 50 in Brazil agreed to participate in this study, which utilized a randomized, crossover, double blind research design. The experiment was conducted over a 5-day period in which each player completed two simulated tennis matches of a 3-hour duration. The players received either a CHO or a placebo (PLA) drinking solution during simulated tennis matches. Athlete’s performance parameters were determined by filming each match with two video cameras. Each player was individually tracked for the entire duration of the match to measure the following variables: (1) games won; (2) rally duration; (3) strokes per rally; (4) effective playing time (%); (5) aces; (6) double faults; (7) first service in; (8) second service in; (9) first return in and (10) second return in.

**Results:**

There were no differences between trials in any of the variables analyzed.

**Conclusions:**

CHO supplementation did not improve tennis match play performance under the present experimental conditions.

## Background

There is strong evidence that appropriate selection of nutrients, timing of intake, and proper supplement choice are associated with optimal health and exercise performance [1]. During exercise, carbohydrate (CHO) supplementation is one of the most popular dietary recommendations to provide energy to skeletal muscles and the central nervous system [1-6]. Further, to ensure proper CHO delivery to the contracting skeletal muscles, the American College of Sports Medicine along with the Academy of Nutrition and Dietetics (AND) (formerly recognized as the American Dietetic Association) each recommend ingestion of a CHO solution during prolonged exercise [1,5]. This recommendation is supported by early empirical evidence regarding the positive effects of CHO supplementation to enhance endurance exercise performance [7,8].

However, even though a tennis match encompasses a long total period of time, the overall exercise requirements of a match differ from traditional endurance exercise. To illustrate, a tennis match involves intermittent bouts of high-intensity effort interspersed with periods of low-intensity activity, during which active recovery (between points) and passive periods (between changeover breaks in play) take place (20 s), over an extended period of time [9-11]. In the major international tournaments (e.g. Grand Slam events and Davis Cup), male players may play several matches within a relatively short period of time (i.e. <2 hours), however, some matches may extend to greater than 5 hours. Consequently, it is conceivable that such a long tennis match may deplete glycogen stores and produce a state of hypoglycemia [12]. Therefore, CHO ingestion may be an interesting approach to avoid significant decrements to a player’s performance. Presently, only a few studies have investigated the effects of CHO supplementation on tennis performance [13-18].

Moreover, the available data regarding the benefits of CHO supplementation on tennis performance are equivocal. For example, hitting accuracy decreased in the PLA trial when compared to the CHO trial [16]. Similarly, CHO supplementation maintained ground stroke accuracy and increased muscle power after simulated tennis tournament [17]. Conversely, a previous study did not observe any significant positive effect of CHO ingestion on serve and ground stroke velocity as well as stroke accuracy during tennis match play [13]. Additional investigations observed similar results showing no significant effect in the CHO condition when compared to a PLA regarding serve velocity or unforced error [14], fan drill speed and percentage points won and lost [15] during tennis match play. In contrast, Ferrauti & Weber [18] reported that CHO supplementation improved tennis specific running speed test, but interestingly this improvement in speed had no effect on stroke accuracy and games won during a match simulation. Ultimately, there have been controversial results regarding the effects of CHO supplementation on tennis performance [13-18], however, the authors of the present investigation hypothesized that CHO supplementation would serve to avoid performance decrement during prolonged tennis match play. Therefore, the aim of the present investigation was to assess the effect of CHO supplementation on tennis match play performance among nationally ranked young players.

## Methods

### Participants

A total of 12 (mean and SD: 18.0 ± 1.0 years; 176 ± 3.4 cm; 68.0 ± 2.3 kg; body fat: 13.7 ± 2.4%), competitive male tennis, involved in regular tennis competitions at the national level, with a national ranking between 10 and 55, volunteered to participate in this study. The mean training background of the players was 15 hoursper week, for a minimum of 5 years. Prior to participation, the experimental procedures and potential risks were fully explained to the athletes and their parents. Additionally, written informed consent was obtained from both the players and their parents. Players with any pre-existing medical conditions (i.e. musculoskeletal injuries, metabolic disorders, severe illness) that could have influence in their hormonal responses or performance were excluded from the study. The study protocol was approved by the Human Subject Committee of the University of São Paulo, CAAE: 09860412.6.0000.5391.

### Experimental design

**Figure 1:**
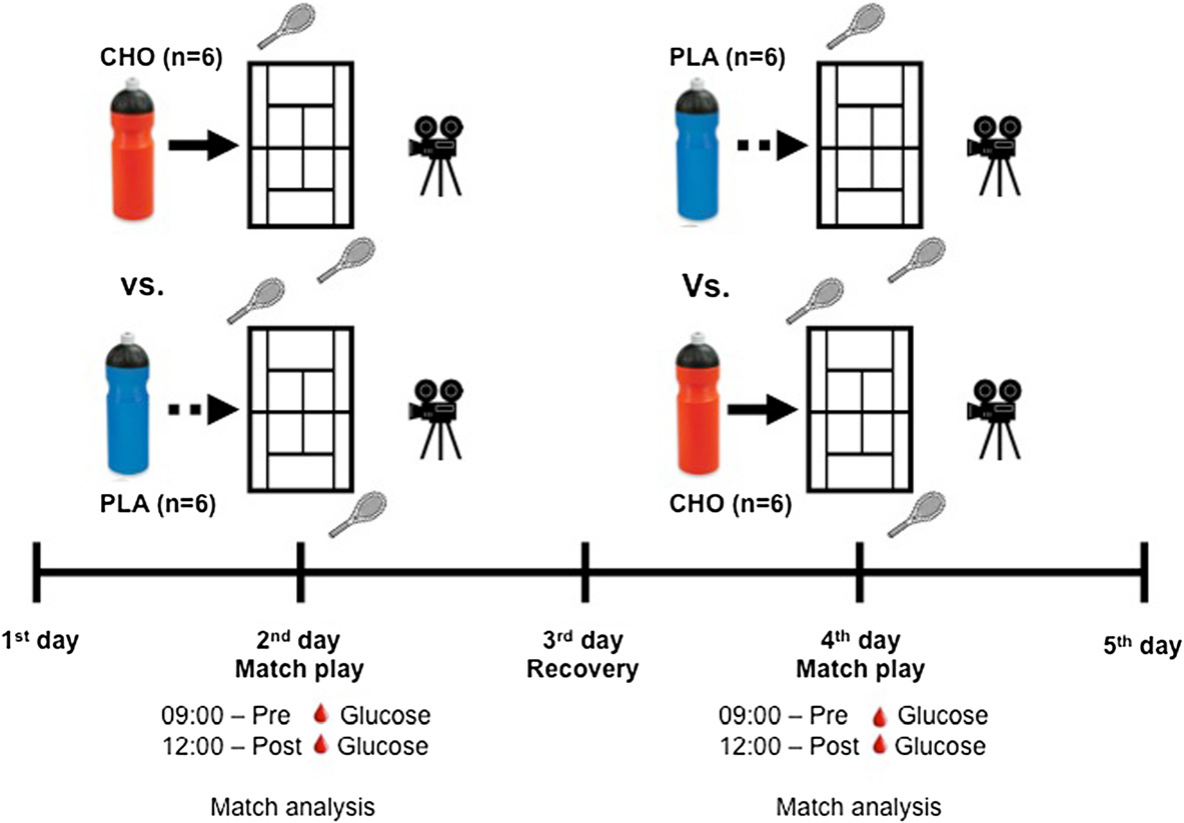
Experimental design.

This study was conducted over a 5-day period, in which each player completed 3 hours of simulated tennis match play, on 2 separate occasions (Figure 1). Subjects ingested either a CHO or PLA beverage in a double blind, randomized, placebo-controlled crossover design. A 48-hour recovery period was granted between each match. During the 2 weeks prior to commencing the study, all players decreased training volume (from 2 sessions to 1 session per day) to ensure each athlete was properly recovered at the study’s onset. Additionally, this study was conducted during a training camp and researchers carefully controlled food and fluid ingestion, and exercise volume.

With the exception of the modified match structure, all games were played according the rules of the International Tennis Federation (ITF) [19] and conducted on an outdoor red clay court. Following the ITF rules, tennis balls (Fort Clay Court Dunlop^©^, Philippines) were replaced with new balls after the 7th game of each set and again every nine games afterward. Each match was structured so that each 'set’ lasted 1 hour, regardless of the current score. Additionally, players competed against a different opponent each set to ensure a similar competition stimulus during each simulation. The athletes were divided into 3 groups of 4 players, and were allocated the same opponents in the same order (i.e. 1st hour: Player A vs. Player B; Player C x Player D, 2nd hour: Player A vs. Player C, Player B vs. Player D, and 3rd hour: Player A vs. Player D; Player C vs. Player B) for both conditions. The player who won the greatest number of points in each set was considered the winner. Each match was officiated by a qualified tennis referee, who also annotated points won by each player. Finally, the athletes were instructed to put maximal effort in both matches.

Finally, before each match, players performed a standardized warm-up, which consisted of 5-min of groundstrokes, volleys + overheads, and serves. The ambient temperature (day 1: 22.0 ± 1.8°C and day 2: 22.6 ± 1.5°C) and relative humidity (day 1: 77 ± 3% and day 2: 75 ± 2%) were similar between testing days.

### CHO supplementation

The sequence of CHO or PLA conditions was randomized as part of the double-blind, crossover study design. At the start of each hour, all athletes ingested either a bottle of CHO solution (6%) containing maltodextrin or water artificially sweetened to comprise the PLA. Thus, players ingested three bottles during each 180-minute match (1 bottle per h - 0.5 g · kg^-1^·h^-1^) [1,4,20]. The solutions were similar in taste and served chilled in opaque containers. Once these solutions were consumed, players were allowed to drink water ad libitum from individually labelled bottles of known volumes. All of the subjects consumed the total volume of the experimental solution in both the CHO and PLA conditions. Therefore, the total volume of fluid consumption was similar between trials (CHO vs. PLA).

During the 24 hours prior both trials, players consumed an isoenergetic-diet prepared by a sports dietitian (CHO: 8.33±0.58 g · kg^-1^; Protein: 2.10±0.14 g · kg^-1^; Fat: 1.58±0.13 g · kg^-1^). Additionally, before each match (7:30), subjects received a standardized CHO solution (Maltodextrin solution; 1 g · kg^-1^; 10%). The daily energy expenditure of athletes was estimated from the following steps:

a) Basal energy expenditure (BEE) was estimated in accordance with the World Health Organization

b) Diet induced thermogenesis was calculated as 10% BEE, and

c) Energy expenditure for physical activity was estimated using metabolic equivalents (METs) [21].

### Match analysis

The activity profile (specific measures of this profile are described later in this section) of each match was determined by filming the matches with two video cameras (DCR-HC17E, Sony^©^, Japan) positioned 2 meters away from the side of the court, at the level with the service line and approximately 6 meters above the court. Each player was individually 'tracked’ to record for the activity profile measures for the entire duration of each match. The video recordings were replayed on a monitor to measure each player’s activity profile in detail. The same researcher performed the video analysis of each player’s activity profile. A modified match analysis protocol developed by Smekal *et al.*[22] was used to extract the following information as variables of a tennis match to comprise the activity profile: 1. games won; 2. rally duration (seconds); 3. strokes per rally; 4. effective playing time (%); 5. aces; 6. double faults; 7. first service in; 8. second service in; 9. first return in and 10. second return in. The validity and reliability of this protocol has been previously described in the literature [23].

Match analysis included (1) rally duration (s); (2) strokes per rally; (3) effective playing time (%). Rally duration was recorded from the time the service player served the first ball until the moment when one of the players won the point. Strokes per rally were quantified as the number of balls hit by the players from the first serve in to the end of the point. Therefore, for rally duration and strokes per rally, the time for first serve faults, as well as the stroke for the serve fault, and the time between first and second service were excluded from the analysis. Effective playing time was defined as the real playing time (sum of all the rally durations) divided by the total match duration multiplied by 100, as described by Fernandez-Fernandez *et al*. [9].

### Blood glucose

Glycemia was determined from a blood sample drawn from the ear lobe and analyzed in the Accu-Chek^©^ monitor (Accu-Chek Active, Roche^©^, Germany). This method of analysis is in accordance with a previous study, which categorized this monitor as “clinically accurate” [24]. Blood samples were drawn while the players were seated prior to and immediately after the matches. The glycemia test-retest had a coefficient of variation (CV) of 3.1%.

### Statistical analyses

All variables were checked for normal distribution and extreme observations using standard procedures. Blood glucose level was analysed using linear mixed models having condition (i.e. CHO and PLA) and time (i.e. Pre and Post) as fixed factors and subjects as a random factor. Whenever a significant F-value was obtained a post-hoc test with a Tukey adjustment was performed for multiple comparison purposes. Match analysis variables were analysed using paired t-test with Bonferroni correction for multiple comparisons. Significance level was set at p<0.05.

## Results

### Blood glucose

**Figure 2:**
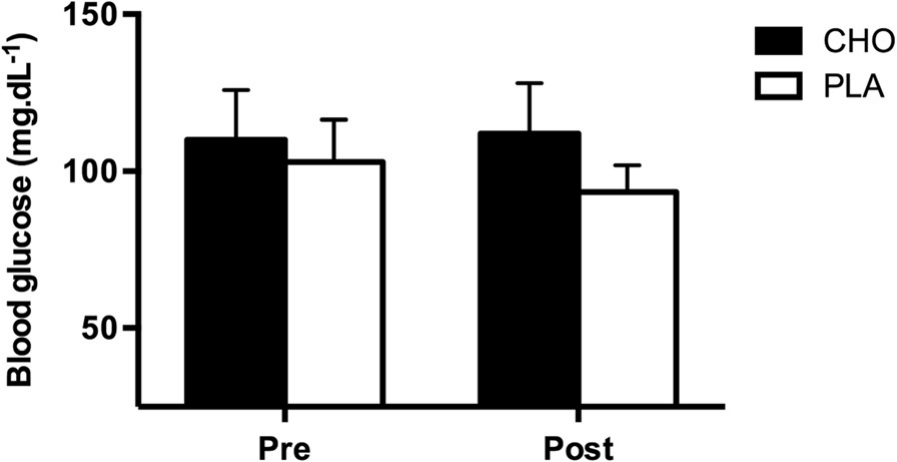
Blood glucose concentration (mean±SD) during PLA and CHO conditions.

There were no significant changes in blood glucose between conditions and from pre- to post-match. However, blood glucose in the CHO condition approached significance (p = 0.06) to being higher (113.4±18.0 mg · dL^-1^), when compared to PLA (93.6±9.0 mg · dL^-1^) (Figure 2), at the end of the tennis match play.

### Match analysis

**Figure 3:**
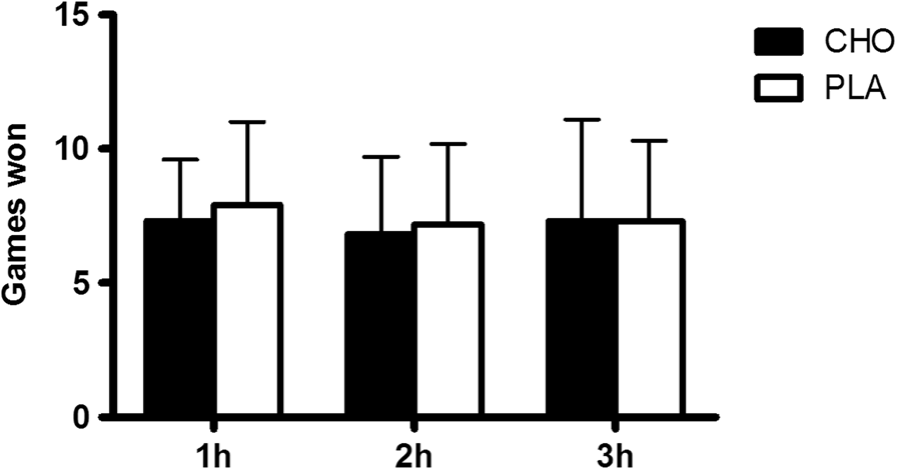
Sum of games won between PLA and CHO conditions.

**Figure 4:**
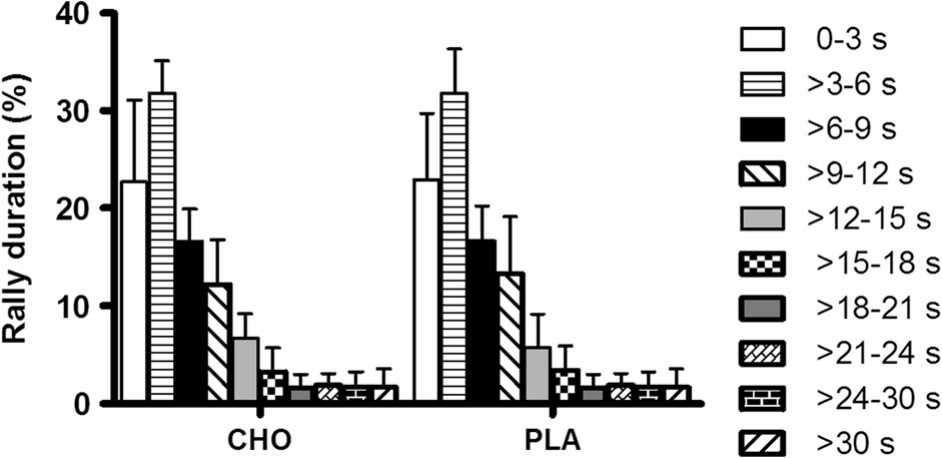
Distribution of rallies duration (%; mean±SD) during PLA and CHO conditions.

**Figure 5:**
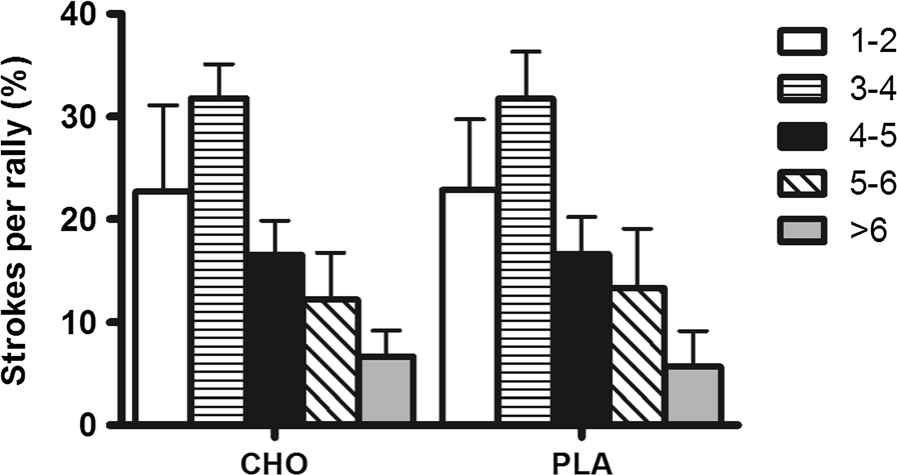
Distribution of strokes per rally (%; mean±SD) during PLA and CHO conditions.

Match analysis of the activity profile revealed no significant differences in the number of games won between conditions (Figure 3). Similarly, there were no differences in rally duration (Figure 4) and number of strokes per rally (Figure 5) between the CHO supplementation and PLA conditions. Additionally, there were no differences in all parameters evaluated between conditions (first service in; second service in; first return in; second return in and baseline return in) (Table 1). Finally, effective playing time was (CHO: 19.1% and PLA: 19.3%), and the number of aces and double faults were similar between experimental conditions (Table 2).

**Table 1:** Technical tennis match play analysis (%; mean±SD) during PLA and CHO conditions

	**%**					
	**1**^**st**^**hour**		**2**^**nd**^**hour**		**3**^**rd**^**hour**	
	**CHO**	**PLA**	**CHO**	**PLA**	**CHO**	**PLA**
First serves in	57±8	53±12	59±8	60±9	61±10	58±11
Second serves in	75±8	82±10	80±15	80±9	87±11	81±12
Return first serve in	70±19	79±12	74±14	73±12	73±18	75±18
Return second serve in	68±9	83±12	75±17	82±16	80±20	82±19
Return first serve in (Forehand)	69±17	76±13	76±17	71±20	74±17	75±13
Return first serve in (Backhand)	71±23	84±21	74±14	73±19	62±23	69±17
Return second serve in (Forehand)	72±9	85±6	74±12	82±12	78±8	74±10
Return second serve in (Backhand)	70±15	71±8	81±4	86±7	83±10	95±8
Baseline return in (Forehand)	75±8	78±4	76±8	76±8	67±10	71±12
Baseline return in (Backhand)	71±10	75±7	71±8	75±7	74±13	73±11

**Table 2:** Number of aces and double faults during PLA and CHO conditions

	**1**^**st**^**hour**		**2**^**nd**^**hour**		**3**^**rd**^**hour**	
	**CHO**	**PLA**	**CHO**	**PLA**	**CHO**	**PLA**
Aces	4.0±1.4	3.8±1.5	3.5±1.2	2.9±1.2	3.7±1.2	3.2±1.1
Double faults	4.9±3.3	4.4±3.5	3.5±2.3	3.7±2.5	2.3±2.1	3.1±2.1

## Discussion

The purpose of this investigation was to assess the effects of CHO supplementation on variables related to match play performance in young tennis players. The main finding of the present study was that CHO supplementation did not affect match play performance variables or have a statistically significant effect on blood glucose level. However, blood glucose in the CHO condition showed a trend toward higher concentration as the statistical analysis approached significance (p = 0.06).

In agreement with the present results, CHO supplementation has been shown to have no effect on tennis match play performance [13-15]. However, previous research has also demonstrated that CHO supplementation is beneficial for improving elements of tennis match play such as stroke performance (accuracy and consistency) [16,17,25] as well as jumping and sprinting performance following a match [17,18]. It should be noted however, that the improvement of stroke accuracy or consistency in a well-controlled research setting may not represent the practical challenges during an actual tennis match play, which include serious tactical, technical and psychological challenges and components. Similarly, although improvements in jumping and sprinting are related to greater anaerobic power, it is not certain that these benefits in a research setting will directly translate to a better match play performance.

The effects of CHO supplementation on exercise performance are associated with the maintenance of blood glucose and the sparing of muscle glycogen stores through the exercise duration [2,3,6,20,26]. However, the results of the present study reveal no significant difference in blood glucose level between PLA and CHO conditions. A possible explanation for the lack of difference in blood glucose level may be that the present study design simulated match play performance, possibly causing the athletes to have a higher sympathetic activity compared with traditional laboratory settings [27]. The hepatic and pancreatic sympathetic activation causes an increased glucose output from the liver as well as a stimulation of glucagon secretion and an inhibition of insulin release from the pancreas [28,29]. Thus, it is reasonable to suggest that interplay of these factors could have prevented the fall of the blood glucose observed in the present study.

Further analysis unravels that the presented findings are consistent with the suggestion of Mitchell *et al.*[14] who note that blood glucose concentration in tennis players may remain stable for up to 180 min of match play. In additional corroboration to the results of the present study, Bergeron *et al.*[30] demonstrated that blood glucose was not significantly decreased following 85 minutes of match play.

Conversely, previous research does exist that prolonged strenuous exercise decreases blood glucose [6,20], and glycogen stores [26] suggesting the necessity of CHO supplementation for similar exercise activities and possibly sports with requirements of intermittent high intensity bouts. For instance, Curell *et al.*[31] reported that CHO supplementation improved performance in 90 minutes of soccer performance test and Winnick *et al.*[32] observed improvements in physical and central nervous system (CNS) functioning tests while mimicking intermittent sports. These studies, however, were in a laboratory setting and did not replicate an actual match or game, thus as suggested earlier it is possible that sympathetic activity was not elevated to same degree during the simulated match play in the present study.

To further explain the absence of difference in blood glucose between conditions, it has been reported that as exercise intensity increases CHO oxidation increases as well lowering blood glucose [33]. To illustrate, Gomes *et al.*[34] reported no significant change in blood glucose level following prolonged tennis match play (197 min), which was accompanied by an increase in blood cortisol. This maintenance of blood glucose with an increased cortisol concentration is quite possibly associated with the activation of gluconeogenesis and glycogenolysis [35]. These factors suggest the possibility that cortisol release might activate gluconeogenesis eliciting the maintenance of blood glucose. Ultimately, the lack of difference in blood glucose between conditions yielded similar patterns of performance during both trails (CHO vs. PLA). Therefore, it is possible that the metabolic demands of tennis are not sufficient to significantly alter blood glucose during tennis match play to warrant supplementation with CHO [14].

Even though CHO supplementation is often used to spare muscle glycogen stores during prolonged exercise, as performance seems to be impaired by low CHO availability [2,3,20,26,36] that did not seem to be the case in the present study. However, prolonged exercise (> 90 min at 55–75% of maximum oxygen uptake - VO_2max_) does seem to decrease blood glucose and muscle glycogen stores [20,26]. Therefore, it is worth noting that as the results of the present investigation demonstrated a trend toward higher blood glucose level in the CHO condition, one may speculate that decrement in blood glucose concentration could reach significance during a second match performed with less than 24 hours of rest interval, leading to deleterious performance effects. These data, make it is reasonable to presume that CHO supplementation may be beneficial to maintain blood glucose level and augment performance under tournament conditions (i.e. ATP, Challengers, Future and national tournaments), when matches are performed within 24 hours as a moderate impairment of glycogen stores during the initial match may cause a drop in blood glucose in the subsequent match [12].

CHO supplementation during exercise may have several benefits including an attenuation in central fatigue; a better maintenance of blood glucose and CHO oxidation rate an improved muscle glycogen sparing effect; a reduced exercise-induced strain; and a better maintenance of excitation-contraction coupling [36]. The maintenance of blood glucose might delay fatigue by attenuating the rise in free fatty acids. This process may convincingly limit the increase of precursors related to central fatigue (i.e. serotonin) [37,38]. Further, support is mounting to provide a link between reduced neural drive to skeletal muscle and fatigue throughout tennis matches [39-41]. Moreover it has been suggested that CHO supplementation may increase neural drive thus leading to an attenuation of fatigue and increased exercise performance [36].

A limitation of the present was that the protocol simulated tennis match play conditions, however, it did not simulate tournament conditions in which athletes would play multiple matches with short recovery periods. Thus, the presented findings cannot be extrapolated to tournament conditions that include multiple matches. A second limitation is that athletes received a high CHO diet (~60% daily energy expenditure; 8.33 g · kg^-1^ · day^-1^) during the experiment, which may have diminished the need for exogenous ingestion of CHO during the tennis match play. It is likely, that the high CHO diet and the rest period between matches (48 hours) was an ample protocol to fill glycogen stores, explaining the maintenance of blood glucose observed in the PLA condition. However, it is important to mention that previous investigations have reported that athletes do not achieve the daily CHO intake recommended during training and competitions [2,42] and as a result liver and muscle glycogen stores might be compromised. In such scenario, CHO supplementation could be alternative to provide energy and spare glycogen stores, delaying fatigue and attenuating performance decrement. Finally, the results of the present study should be interpreted with caution considering that the study’s sample consisted of well-trained athletes, who might have advanced physiological adaptations that could modulate the responses observed (e.g. more efficient counter-regulatory hormonal response, greater hepatic glucose production, lower reliance on carbohydrates and higher utilization of lipids as energy substrate [43]), which may not otherwise occur in a less-advance athletic population.

## Conclusions

The main finding of the present study were: first, CHO supplementation does not augment measures of tennis match play performance and, second, no significant difference in blood glucose was detected after CHO trial compared to a PLA during 180 minutes of simulated match play, however there was a trend toward higher blood glucose in the CHO trial. It is possible that the metabolic demands of 180 minutes of tennis match play are not great enough to significantly lower blood glucose when players were fed a sufficient CHO diet (>8 g · kg^-1^·day^-1^). However, during prolonged matches or tournaments that require multiple matches in a 24-hour time span an athlete may benefit from CHO supplementation. Therefore, coaches and athletes should carefully assess the timing and requirements of a single match or a tournament and determine if CHO supplementation is necessary. Further research is necessary to investigate the effects of CHO supplementation during longer matches and in tournament-style play of multiple matches in a 24-hour time span to clarify recommendations.

## Competing interests

The authors declare that they have no competing interests.

## Authors’ contributions

RVG was responsible for data collection, data analysis and interpretation, and the writing of the draft. CDC helped with data collection and contributed to data analysis and interpretation. CU helped with statistical analysis and writing of the manuscript. MCZ participated in data analysis and the writing of the manuscript. JFF and AMV helped in data analysis and interpretation. MSA designed the study and supervised the data collection, analysis, and helped with the writing of the manuscript. All authors read and approved the final manuscript.
